# High-temperature superconductivity up to 223 K in the Al stabilized metastable hexagonal lanthanum superhydride

**DOI:** 10.1093/nsr/nwad107

**Published:** 2023-04-20

**Authors:** Su Chen, Yingcai Qian, Xiaoli Huang, Wuhao Chen, Jianning Guo, Kexin Zhang, Jinglei Zhang, Huiqiu Yuan, Tian Cui

**Affiliations:** State Key Laboratory of Superhard Materials, College of Physics, Jilin University, Changchun130012, China; High Magnetic Field Laboratory, Hefei Institutes of Physical Science, Chinese Academy of Sciences, Hefei230031, China; State Key Laboratory of Superhard Materials, College of Physics, Jilin University, Changchun130012, China; State Key Laboratory of Superhard Materials, College of Physics, Jilin University, Changchun130012, China; State Key Laboratory of Superhard Materials, College of Physics, Jilin University, Changchun130012, China; State Key Laboratory of Superhard Materials, College of Physics, Jilin University, Changchun130012, China; High Magnetic Field Laboratory, Hefei Institutes of Physical Science, Chinese Academy of Sciences, Hefei230031, China; Center for Correlated Matter, College of Physics, Zhejiang University, Hangzhou 310058, China; School of Physical Science and Technology, Ningbo University, Ningbo315211, China; State Key Laboratory of Superhard Materials, College of Physics, Jilin University, Changchun130012, China

**Keywords:** superhydride, metastable, high pressure, superconductivity

## Abstract

As compressed hydrides constantly refresh the records of superconducting critical temperatures (*T_c_*) in the vicinity of room temperature, this further reinforces the confidence to find more high-temperature superconducting hydrides. In this process, metastable phases of superhydrides offer enough possibilities to access superior superconducting properties. Here we report a metastable hexagonal lanthanum superhydride (*P*6_3_*/mmc*-LaH_10_) stabilized at 146 GPa by introducing an appropriate proportion of Al, which exhibits high-temperature superconductivity with *T_c_* ∼ 178 K, and this value is enhanced to a maximum *T_c_* ∼ 223 K at 164 GPa. A huge upper critical magnetic field value *H_c_*_2_(0) reaches 223 T at 146 GPa. The small volume expansion of *P*6_3_*/mmc*-(La, Al) H_10_ compared with the binary LaH_10_ indicates the possible interstitial sites of Al atoms filling into the La–H lattice, instead of forming conventional ternary alloy-based superhydrides. This work provides a new strategy for metastable high-temperature superconductors through the multiple-element system.

## Teaser

This work provides a new strategy to obtain the metastable lanthanum superhydride with excellent superconductivity by Al introduction with appropriate content.

## INTRODUCTION

Metastable compounds refer to a category of compounds characterized by dynamic stability and higher formation energies in thermodynamics, and have always been a hot topic. Based on data from the Inorganic Crystal Structure Database, ∼20% of experimentally synthesized materials exhibit metastable behavior. Among these materials, some display high positive formation enthalpies, resulting in energy gains of >50 meV/atom [1–3]. The discovery and synthesis of new metastable phases are promising avenues for innovations in materials science. Until now, many important achievements have been realized in the structure and superconductivity of hydrides. The discovery of high-temperature superconductivity (HTS) in sulfur hydride has triggered the rush in hydrogen-based superconductors [4–7]. We note that some of these hydrides are metastable compounds, such as the theoretically predicted room temperature superconducting superhydride YH_10_ [8], ‘thermal conductivity’ hydride Li_2_MgH_16_ [1], ‘pentagon-like’ HfH_10_ [9] and experimentally obtained hexagonal structures PrH_9_ [10] and NdH_9_ [11], etc.

The high-*T_c_* phases of lanthanum hydrides deserve special attention because this system holds the highest *T_c_* in binary hydrides [12–15]. Somayazulu *et al.* discovered the HTS of LaH_10±x_ (–1 ≤ x ≤ 2) with *T_c_* ∼ 260 K at 188 GPa [12] and the X-ray diffraction (XRD) data were consistent with the LaH_10_ sample reported by the experimental synthesis [13]. Later, the data provided by Drozdov *et al.* also confirmed the superconducting response of cubic *Fm*$\bar{3}$*m*-LaH_10_ phase with *T_c_* ∼ 252 K at 170 GPa [14] and Hong *et al.* obtained the possible similar superconducting phase with *T_c_* ∼ 240 K [15]. Recently Sun *et al.* reported that the *Fm*$\bar{3}$*m* phase of LaH_10_ can be stabilized at significantly lower pressures (136 GPa) with *T_c_* of 246 K [16]. Besides the cubic LaH_10_ phase, multiple distorted phases of LaH_10_ are predicted in theory as well. A new hexagonal *P*6_3_/*mmc*-LaH_10_ phase predicted by Shipley *et al.* was stable at 420 GPa [17], which was also another potential high-temperature superconductor with *T_c_* > 200 K. Although the experiments of the binary lanthanum hydrides have triggered the sign of this phase [14,16], as the impurity is accompanied by the emergence of the main cubic phase, further details of this phase and corresponding superconductivity have not been obtained. As is known to all, metastable phases with higher formation energies may be obtained by using high-pressure, high-temperature and/or non-equilibrium conditions [18]. One strategy to lower the stabilization pressure or increase the *T_c_* is the introduction of the other elements that have been successfully applied to the cuprate-based and iron-based superconductors [19–22]. Likewise, the idea of introducing dopants under pressure has been used in systems to predict hydrides [23,24]. Recently, a new set of ternary hydrides (with stoichiometry AXH_8_) were proposed to become HTS at moderate pressures [25]. This motivates the desire to obtain these metastable and high-temperature superconducting lanthanum-based hydrides in the experiment.

In this paper, we demonstrate a strategy to decrease the stabilization pressure of the binary lanthanum hydrides with high *T_c_*, through the construction of ternary hydrides. By the introduction of Al with a smaller atomic radius, we find that the synthesized *P*6_3_*/mmc* LaH_10_ phase becomes stable in the pressure range of 146–183 GPa rather than the cubic phase. The four-probe electrical resistance measurements detect the HTS in this ternary superhydride with a maximum *T_c_* ∼ 223 K. These results display that the introduction of Al lowers the formation enthalpy of *P*6_3_*/mmc-*LaH_10_. The small volume expansion of the La–Al–H system compared with the binary La–H system indicates the possible interstitial sites of Al atoms filling into the La–H lattice, instead of forming conventional alloy-based ternary systems. The realization of this compound is a bit unusual in contrast to previously reported ternary hydrides.

## RESULTS AND DISCUSSION

### Superconductivity in the La–Al–H system

**Figure 1.**
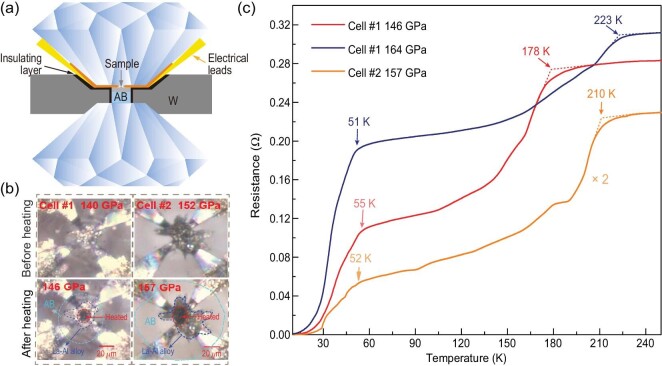
Sample chamber and superconducting transitions in the synthesized La–Al–H sample under high pressure. (a) Schematic of the electrical transport measurements. (b) Optical micrographs of the sample chamber in Cell #1 and Cell #2 before and after laser heating. (c) Typical temperature dependence of resistance in Cell #1 and Cell #2 at typical pressures during the cooling process. The ratio of the initial La–Al alloy in Cell #1 and Cell #2 is La_0.8_:Al_0.2_. The arrows indicate the *T_c_*s.

In this work, we have chosen ammonia borane (NH_3_BH_3_, AB) as both a hydrogen source and a pressure transmitting medium. Its decomposition reaction is NH_3_BH_3_→3H_2_ + c-BN [26,27] and this approach has been proven effective in previous studies [11,28,29]. In order to investigate the potential superconductivity of the La–Al–H system at high pressure, we have prepared a series of La–Al alloy by using magnetron sputter deposition and performed seven experimental runs (Cells #1–7). Three different ratios for La–Al alloy have been obtained for Cells #1–7, with ratios of 0.8:0.2, 0.9:0.1 and 0.7:0.3 (Supplementary Table S1). Through these experimental settings, we have acquired more information about the effect of Al atoms introduced into the La–H system. The uniform parts of the La–Al alloy were selected and loaded into the diamond anvil cell (DAC) together with AB. The schematic set-up of the electrical transport measurements is shown in Fig. 1a. Details of the experimental parameters are available in the Supplementary information. All the prepared cells were laser heated to ∼1500 K at the target pressures and the heated part of the sample obviously turned black due to the reaction with hydrogen (Fig. 1b). In Cell #1, the pressure was increased from 140 to 146 GPa after laser heating. From the measurements of the electrical resistance, evidence for two superconducting transitions was observed with *T_c_*_1_ ∼ 178 K for the high-*T_c_* phase and *T_c_*_2_ ∼ 55 K for the low-*T_c_* phase, arising from the disparate phases as shown in the next section. When the pressure was increased to 164 GPa, *T_c_*_1_ increased to a maximum value of 223 K (Fig. 1c). Two similar resistance drops can be clearly seen in Cell #2 at 157 GPa with 210 and 52 K, respectively. These two cells with the same ratio for La–Al alloy displayed similar HTS transitions. In Cells #3 and #4, with low Al content (10%), we found that the superconducting phase displayed the highest *T_c_* of 120 K after multiple heating (Supplementary Figs S12 and S19). This superconducting phase was further verified by the application of the external magnetic field (Supplementary Fig. S13b). This superconducting phase with much lower *T_c_* than the *P*6_3_*/mmc*-(La, Al) H_10_ phase possibly suggests that the content of Al plays an indispensable role in the formation of the HTS ternary La–Al–H system.

Besides, we also have tried to achieve the same La–Al ratio as the theoretical work reported such as a 1:1 La–Al sample. Unfortunately, when the Al content in the initial alloy is higher, the synthesized sample is inclined to form the semiconducting phase or some phases with lower hydrogen content. We have checked the other samples with higher Al content (30%) both in Cells #5 and #6. In Cell #5, after heating, the resistance of the sample increased by 100-fold and the resistance increased with decreasing temperature showing the semiconductor characteristics (Supplementary Fig. S25). After multiple heating at a higher pressure of 119 GPa, XRD characterization revealed a mixed phases of several La–Al hydrides with low hydrogen content (Supplementary Fig. S26). We also attempted to heat this sample at a higher pressure of 157 GPa in Cell #6 but the electrical measurements still showed its semiconducting properties (Supplementary Fig. S28). Recent theoretical work has shown that most elements distort or even destroy H cages at higher doping levels [23]. Considering that the introduction of different proportions of Al under similar synthesis conditions results in drastic changes in *T_c_*, we think that the introduction of Al possibly leads to the deformation of the H cages and the degree of deformation will vary with the introduced ratio. At the same time, the electronic singularity may be greatly affected, resulting in a difference in *T_c_*. When the Al content introduced reaches ∼0.3, the hydrogen cages are destroyed, inducing big changes in the structure, and thus may form more stable low-ratio hydrides. The present results further indicate that the content of Al in the samples has an optimal value for the emergence of HTS with *T_c_* > 200 K (Supplementary Fig. S31) but further information needs more experiments with different ratios of La–Al samples.

**Figure 2.**
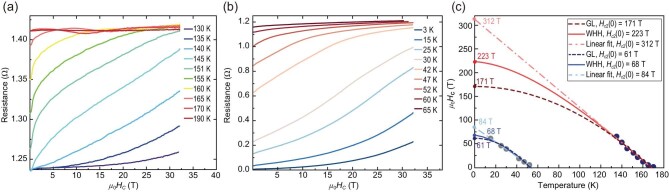
Upper critical fields as a function of the temperature for the La–Al–H sample in Cell #1. Resistance as a function of the field *μ*_0_*H_c_* under the continuous field for the (a) *T_c_*_1_ phase and (b) *T_c_*_2_ phase at 146 GPa in Cell #1. Here, *H_c_*_2_ is defined as the intersection between a line having the slope of the resistive transition and an extrapolation of the resistance in the normal state. (c) The upper critical magnetic field is fitted by three models. Blue and gray circles denote the values of *H_c_*_2_ of two different phases in (a) and (b). Solid lines, dashed lines and dotted lines correspond to the WHH [30], GL [31] and linear [32,33] models fitting to the experimental data.

The superconducting transitions of the La–Al–H sample at 146 GPa are further verified by measuring the isothermal resistance as a function of the external magnetic field *μ*_0_*H_c_* at the selected temperature range in Cell #1 (Fig. 2). In this work, we report measurements of the upper critical field [*H_c_*_2_(0)] in the La–Al–H sample under extremely high magnetic fields of ≤32 T. To make it clear, Fig. 2a and b displays the field-dependent resistance at various temperatures for the two superconducting phases, respectively. One can see that the electrical resistance is eventually enhanced by applying the magnetic fields, confirming the nature of the superconducting transitions. However, the superconducting states at the lower temperatures are not fully suppressed at the maximum magnetic field of 32 T, suggesting the very large upper critical field *H_c_*_2_(0) for this system. In Fig. 2c, the upper critical field *H_c_*_2_(0) estimated from the extrapolation of the resistive transition and the normal state values are fitted by using the Werthamer–Helfand–Hohenberg (WHH) model [30], Ginzburg–Landau (GL) model [31] and a linear model [32,33], which gives *H_c_*_2_(0) = 223 T (68 T), 171T (61 T) and 312 T (84 T) for the high-*T_c_* (low-*T_c_*) phase, respectively. Furthermore, for the WHH model, the corresponding coherence length ξ (0) in the high-*T_c_* (low-*T_c_*) phase is 1.2 nm (2.2 nm). The Pauli limit is 1.86 × *T_c_*, which corresponds to 331 T (102 T) for the high-*T_c_* (low-*T_c_*) phase, and the extrapolated *H_c_*_2_(0) in this experiment differs from the Pauli limit by a factor of ∼1.5, thus indicating that this high-*T_c_* (low-*T_c_*) phase is an orbital-limited superconductor, which is similar to *Fm*$\bar{3}$*m*-H_3_S [32], *C*2*/m*-LaH_10_ and *Fm*$\bar{3}$*m*-LaH_10_ [16]. To further understand the superconducting behaviors in the high-*T_c_* phases, from *ξ* = 0.18ℏ*υ*_F_/*k*_B_*T_c_* within the BCS theory [34], we obtained the Fermi velocity *υ*_F_$\approx $$1.8 \times {10}^5$ m/s, and from the relationship *υ*_F_ =$\sqrt {\frac{{{\hbar }^2{\mathrm{\ }}}}{{m_e^2}}{{( {3{\pi }^2n} )}}^{2/3}} \ $, the carrier density is estimated to be *n*$\approx $$1.29 \times {10}^{26}$ m^−3^; thus, the London penetration depth can be estimated as λ(0) = $\sqrt {\frac{{{\varepsilon }_0{m}_e{c}^2}}{{n{e}^2}}} $$\approx $ 468 nm, which yields the GL parameter as κ = λ(0)/ξ(0) $\approx $ 334, indicating that this phase is possibly a strong type II superconductor [35].

For both phases, *H_c_*_2_(*T*) shows nearly linear dependence *H_c_*_2_∼ ${|}$*T*−*T_c_*${|}$, which is much closer to the experiment at low temperature in contrast to the WHH model. For conventional Bardeen–Cooper–Schrieffer-type superconductors such as HTS hydrides, the WHH model based on the single-band model is widely used [16,28,36,37]. In this case, we cannot decide which model could fit the data better. In repeated Cell #2, with the same ratio initial La–Al alloy, the *H_c_*_2_(0) estimated by using the WHH model gave 226 T at 157 GPa (Supplementary Fig. S6), consistently with the results of Cell #1. Ultimately, no matter which fitting model is used, the obtained *H_c_*_2_(0) of the high-*T_c_* phase in the La–Al–H system is much larger than those reported in high-temperature superconductors *Fm*$\bar{3}$*m*-LaH_10_ (95–136 T at 150 GPa) [14], *Fm*$\bar{3}$*m*-(La, Y) H_10_ (100–135 T at 183 GPa) [38], *C*2*/m*-LaH_10_ (133.5 T at 120 GPa) [16] and *P*6_3_*/mmc*-YH_9_ (103.2 T at 177 GPa) [39]. The enhancement of the upper critical magnetic field may result from the introduction of Al atoms and distortion of the H cages, which could lead to impurities and crystal defects in the material. Such impurities and defects can enhance the intra-band and inter-band scattering, leading to a shorter average free path of electrons, thus significantly increasing the critical field [30,40–42]. In addition, this high *H_c_*_2_(0) in the La–Al–H system compared with the binary La–H system further indicates the successful introduction of Al atoms into the La–H system.

### Crystal structures of superconducting phases

**Figure 3.**
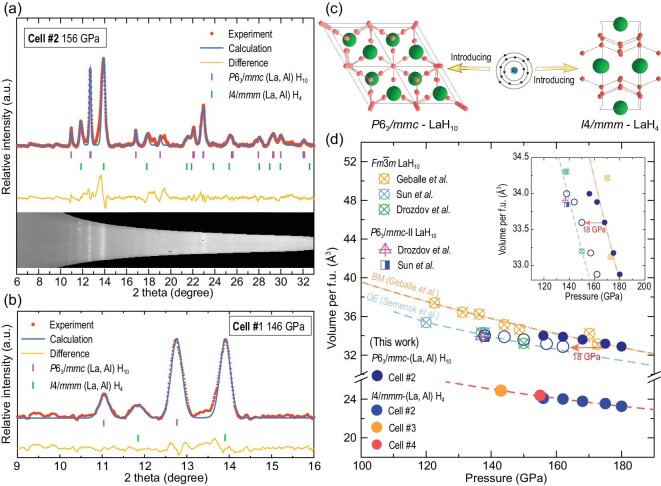
XRD patterns and pressure–volume data of the La–Al–H samples at different pressures. (a) and (b) Synchrotron XRD patterns (λ = 0.6199 Å) and Le Bail refinements of *P*6_3_*/mmc*-(La, Al) H_10_ and *I*4*/mmm*-(La, Al) H_4_ at target pressure for Cell #2 and Cell #1. The experimental data, calculated data and differences are shown in red points, blue lines and yellow lines, respectively. (c) The schematic diagram for the crystal structures of the La–Al–H system. Green, red and blue balls represent La, H and Al atoms, respectively. (d) Pressure dependences of the unit cell volume (per f.u.). Experimental data in this study are represented by solid symbols with different colors. Orange and blue dashed lines indicate the fitted Birch-Murnaghan (BM) line from [13] and calculated data by Quantum Espresso (QE) from [38]. Inset is a partially enlarged view of the data in Cell #2.

XRD is an important technique that is highly sensitive to the characterization of crystal structures. Considering the disparate superconducting phases in the electrical cells, the simultaneous superconductivity and XRD measurements on the same sample and cell are greatly critical. We have obtained the *in situ* synchrotron XRD patterns on the two Cells #1 and #2. Although the location of hydrogen and aluminum atoms cannot be directly determined from synchrotron XRD patterns, the obtained pressure–volume data could give us important information indirectly. For Cell #2, after measuring the electrical resistance at 157 GPa, this cell was taken to the synchrotron beamline and the XRD patterns were collected. Through the detailed analysis, its experimental XRD patterns could be explained by the *P*6_3_*/mmc*-(La, Al) H_10_ and *I*4*/mmm*-(La, Al) H_4_ phases at 156 GPa, as shown in Fig. 3a. Due to the different DAC devices used between Cell #1 and Cell #2, the angle that can be measured in Cell #1 is much smaller than in Cell #2 (Fig. 3b), so we only obtain two peaks for *P*6_3_*/mmc*-(La, Al) H_10_ in this cell, contributing to the relatively larger error in contrast to other samples (Fig. 3d). Nevertheless, we can still judge from the detected peaks that it is the same phase as Cell #2. Besides, we also observed a relatively pure *I*4*/mmm*-(La, Al) H_4_ phase in Cell #3 and Cell #4 (Supplementary Figs S14 and S20). The refined experimental lattice parameters and volumes of *P*6_3_*/mmc*-(La, Al) H_10_ and *I*4*/mmm*-(La, Al) H_4_ are listed in Supplementary Table S2 and the schematic diagram of crystal structures are shown in Fig. 3c. Upon further compression, more volume data have been collected in Cell #2 (Fig. 3d). Comparing these experimentally obtained cell volumes with previous experimental and theoretical results, we tentatively propose that the ratio of metal to hydrogen in the obtained compound is 1:10. Recently, our group also observed a hexagonal phase for the binary La–H system at 130 GPa, but the exact ratio is unknown [42]. According to the theoretical calculations for the binary La–H system, the *P*6_3_*/mmc*-LaH_10_ structure becomes thermodynamically favorable at pressures above ∼420 GPa; more importantly, this *P*6_3_*/mmc* phase is also considered possibly metastable at low pressures and previous theoretical work has indicated that this phase was lying within the 20-meV/atom of the *Fm*$\bar{3}$*m* phase down to 150 GPa [17]. We calculated the phonon spectrum of *P*6_3_*/mmc*-LaH_10_ phase at 150 GPa and found imaginary frequencies (Supplementary Fig. S32), which suggested that this structure may be dynamically unstable at this pressure. Previous experimental work triggered some hints of this *P*6_3_*/mmc*-LaH_10_ phase with weak XRD peaks, which has been considered as a minor impurity in the synthesized *Fm*$\bar{3}$*m*-LaH_10_ sample [14,16]. Interestingly, in this experiment, due to the introduction of Al atoms, *P*6_3_*/mmc*-LaH_10_ was stabilized as the main phase at 146 GPa and its HTS was also first detected. Impressively, outside the family of cubic hydrides, the *T_c_* of this new superhydride with hexagonal symmetry also exceeds 200 K.

In the reported experimental studies for synthesized superhydrides, the pressure scale is an important factor to affect the determination of crystal structures and stoichiometric ratios. At present, two pressure scales (diamond and hydrogen Raman vibron) are widely used in these experiments. Considering the difference between these two pressure scales with ∼18 GPa, as in the previous work reported by Sun *et al.* [16], we also have demonstrated the pressure–volume data manually by reducing the pressure of Cell #2 with 18 GPa, as is shown in the inset of Fig. 3d. Under the same pressure calibration conditions, we find that the cell volume of *P*6_3_*/mmc*-(La, Al) H_10_ is slightly larger than the cell volume of binary *P*6_3_*/mmc*-LaH_10_ (data from Sun *et al.* [16]) and the difference in the unit cell volume is ∼0.145 Å^3^ at 138 GPa, which is consistent with the ratio of Al in the initial La–Al alloy. In this case, we further confirm that the ratio of La–Al in the synthesized La–Al–H system is 0.8:0.2. For the *I*4*/mmm*-(La, Al) H_4_ phase, we find that it could stabilize at >145 GPa with *T_c_* of 55–68 K, which could possibly arise from the predicted LaH_4_ phase [43]. Therefore, the two phases obtained by using XRD analysis explain the two-step superconducting transitions in both Cell #1 and Cell #2. In addition, the XRD results also indicate that in the ternary La–Al–H system, Al atoms may be randomly distributed in the interstitial spaces of the *P*6_3_*/mmc*-LaH_10_ lattice. In the published La–Y–H work [38], a lower unit cell volume of *Fm*$\bar{3}$*m*-(La, Y) H_10_ was observed compared with the binary *Fm*$\bar{3}$*m*-LaH_10_ when larger La atoms were replaced by Y atoms with smaller size. In our work, the atomic size of Al is much smaller than that of Y, but the resulting unit cell volume is indeed slightly larger than binary *P*6_3_*/mmc*-LaH_10_, with a volume difference of 0.145 Å^3^ at 138 GPa. This phenomenon indicates that the introduction of a small amount of Al does not influence the crystal structure of *P*6_3_*/mmc*-LaH_10_, possibly just dispersed in the interstices of the lattice. Exact structural information for the ternary La–Al–H system with disordered atoms is almost impossible to obtain with current technical means. Accurate determination of the structure of such ternary hydrides awaits in-depth research using advanced diagnostic probes such as nuclear magnetic resonance (NMR) [44,45] and high-pressure neutron diffraction [46].

### The pressure dependences of *T_c_*

**Figure 4.**
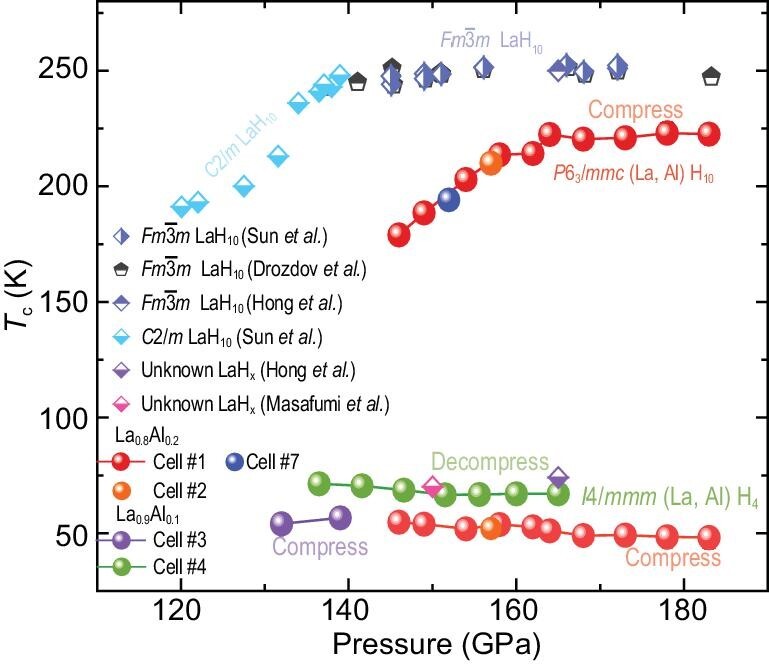
The pressure dependences of *T_c_* for the ternary La–Al–H system and binary La–H system. Experimental *T*_c_ as a function of pressure for synthesized phases with different Al introducing levels in the electrical Cells #1, #2, #3, #4 and #7. The ratio of initial La–Al alloy in Cells #1, #2 and #7 is La_0.8_:Al_0.2_, while in Cells #3 and #4 it is La_0.9_:Al_0.1_. The solid symbols represent the data from this work.

In order to determine the highest value of *T_c_* for La–Al hydrides, we investigated its dependence on pressures, as shown in Fig. 4. The evolution of *T_c_* as a function of pressure was constructed based on the pressure-dependent electrical resistance data in Cells #1–4 and 7. For *P*6_3_*/mmc*-(La, Al) H_10_, it is clearly seen that *T_c_* first increases and later nearly becomes invariant with increasing pressure up to 183 GPa, with a maximum *T_c_* of 223 K at 164 GPa. Besides, the different tendencies between (La, Al) H_10_ and LaH_10_ further confirm that the introduction of Al atoms indeed affects the *T_c_*. However, *I*4*/mmm*-(La, Al) H_4_ exhibits different pressure dependences—that is, *T_c_* decreases very slowly with increasing pressure. Moreover, by comparing the present *T_c_* of *I*4*/mmm*-(La, Al) H_4_ with previous reports, we find that this phase synthesized by Cell #4 is similar to the unknown phase reported by Masafumi *et al.* using AlH_3_ as the hydrogen source [47]. In contrast, the small difference with ∼10 K of *T_c_* for *I*4*/mmm*-(La, Al) H_4_ in Cell #3 and Cell #4 may be due to the different synthesis conditions and the Al introducing levels.

## CONCLUSIONS

In summary, through heating the appropriate La–Al alloys with NH_3_BH_3_ at target pressures, we have successfully synthesized *P*6_3_*/mmc*-(La, Al) H_10_ and *I*4*/mmm*-(La, Al) H_4_ phases in the pressure range of 146–183 GPa. The *P*6_3_*/mmc*-(La, Al) H_10_ phase has a high *T_c_* ∼ 223 K at ∼164 GPa. Besides, by the application of high magnetic fields of ≤32 T, *H_c_*_2_(0) of this phase fitted by using the WHH model reaches the highest value of 223 T at 146 GPa. The small volume expansion of *P*6_3_*/mmc*-(La, Al) H_10_ compared with the binary La–H system indicates the possible interstitial sites of Al atoms filling into the La–H lattice, instead of forming conventional ternary alloy-based superhydrides. Our experiments demonstrate that with appropriate introduction, compounds with high computational stability pressures can be synthesized at lower pressure and this result also provides a promising route for the stability of HTS in superhydrides under mild conditions.

## EXPERIMENTAL METHODS

### Sample preparation

Both lanthanum target (purity 99.9%) and aluminum target (purity 99.999%) were purchased from the Chinese Beijing Hezong New Material Company. A multi-target magnetron sputter system was used for the La–Al alloy deposition. Different ratios of La–Al alloy were deposited on glass substrates by DC/RF co-sputtering at 70–140 W, with pure argon atmosphere and pressure of 0.5 Pa for ∼15 minutes. After sputtering, we quickly transferred the sample to the argon protected glovebox with water and oxygen content of <0.01 ppm. And then we selected a piece of initial La–Al alloy sample and characterized it using scanning electron microscopy (SEM) and energy-dispersive X-ray fluorescence (EDX) analysis. Considering that the SEM + EDS test could possibly oxidize the sample by exposing it to the air for a period, we divided the sample into two parts: one for testing and one for sample loading.

### Electrical transport measurements

The four-probe electrical resistance transport measurements were carried out by using a piston-cylinder DAC made of non-magnetic Ni–Cr–Al or Be–Cu material in a multifunctional measurement system (1.5–300 K, JANIS Research Company Inc.; 0–9 T, Cryomagnetics Inc.). Diamond anvils with a culet of 60–100 μm in diameter beveled 300 μm with an angle of 8° were chosen to generate the ultra-high pressure. Cubic boron nitride (c-BN) and magnesium oxide (MgO) powder mixed with an epoxy binder were used for an insulating layer and tungsten was used as the gasket. Four molybdenum electrodes were sputtered onto the cylindrical diamond surface and then connected to external copper wires by using a 5-μm-thick platinum foil. We chose ammonia borane (NH_3_BH_3_, AB) as both a hydrogen source and a pressure transmitting medium. Its decomposition reaction is NH_3_BH_3_→3H_2_ + c-BN [26,27]. The loaded sample is selected close to the portion characterized by SEM/EDX to ensure consistent proportions. Although the thickness of the sputtered La–Al alloy film is very thin (∼2 μm), the alloy is very soft. When we scrape the sample with a tungsten needle, the alloy will curl together to increase its thickness. Therefore, for every loading, we also pre-pressed the La–Al alloy with the diamond anvils (culet of 400 μm) in an inert Ar atmosphere glovebox, which could reduce the thickness of the sample. The pressure was determined from Raman shift of the diamond edge [48].

### Magnetoresistance measurements

The magnetoresistance measurements were performed at the National High Magnetic Field Lab of China at Hefei. Magnetoresistance was measured using the standard four-probe method in a physical property measurement system and the stable high magnetic field measurements were performed using the standard ac lock-in technique (13.7 Hz) with a He-4 fridge and water-cooling magnet of ≤32 T.

### Structure characterization

*In situ* high-pressure synchrotron XRD measurements were performed at the beamline BL15U1 of Shanghai Synchrotron Radiation Facility (SSRF) with the use of a focused (5 μm × 12 μm) monochromatic beam at a wavelength of 0.6199 Å. The experimental XRD images were integrated and analysed using the Dioptas software package [49]. The full profile analysis of the diffraction patterns and calculation of the unit cell parameters were performed using Materials Studio [50] and Jana2006 [51] programs with the Le Bail method [52].

## Supplementary Material

nwad107_Supplemental_File
