# Supplementary material for: High-temperature superconductivity up to 223 K in the Al stabilized metastable hexagonal lanthanum superhydride
Source: Natl Sci Rev. 2023 Apr 20;11(1):nwad107. doi: 10.1093/nsr/nwad107 (PMC10727841; doi:10.1093/nsr/nwad107)
Supplement: nwad107_Supplemental_File [file nwad107_supplemental_file.docx]

**Supplementary Information**

**for**

**High-temperature superconductivity up to 223 K in the Al stabilized metastable hexagonal lanthanum superhydride**

Su Chen,^1, †^ Yingcai Qian,^2, †^ Xiaoli Huang,^1, *^ Wuhao Chen,^1^ Jianning Guo,^1^ Kexin Zhang,^1^ Jinglei Zhang,^2^ Huiqiu Yuan^3^ and Tian Cui^4, 1, *^

^1^ State Key Laboratory of Superhard Materials, College of Physics, Jilin University, Changchun 130012, China

^2^ High Magnetic Field Laboratory, HFIPS, Anhui, Chinese Academy of Sciences, Hefei 230031, China

^3^ Center for Correlated Matter and Department of Physics, Zhejiang University, Hangzhou 310058, China

^4^ School of Physical Science and Technology, Ningbo University, Ningbo, 315211, China

^†^These authors contributed equally to this work

^*^Corresponding authors, Email: huangxiaoli@jlu.edu.cn (X. Huang), cuitian@nbu.edu.cn (T. Cui)

**Experimental details**

**Table S1.** Additional parameters of the DACs.

| Cell | Culet size (μm) | Gasket | Composition | La:Al | Pressures (GPa) | Measurement |
| --- | --- | --- | --- | --- | --- | --- |
| #1 | 60 | W+MgO/epoxy | LaAl alloy+NH_3_BH_3_ | 0.8:0.2 | 146-183 | SC, XRD |
| #2 | 60 | W+MgO/epoxy | LaAl alloy+NH_3_BH_3_ | 0.8:0.2 | 156-180 | SC, XRD |
| #3 | 60 | W+MgO/epoxy | LaAl alloy+NH_3_BH_3_ | 0.9:0.1 | 132-151 | SC, XRD |
| #4 | 60 | W+MgO/epoxy | LaAl alloy+NH_3_BH_3_ | 0.9:0.1 | 108-165 | SC, XRD |
| #5 | 100 | W+MgO/epoxy | LaAl alloy+NH_3_BH_3_ | 0.7:0.3 | 52-119 | SC, XRD |
| #6 | 60 | W+MgO/epoxy | LaAl alloy+NH_3_BH_3_ | 0.7:0.3 | 157 | SC |
| #7 | 60 | W+MgO/epoxy | LaAl alloy+NH_3_BH_3_ | 0.8:0.2 | 152 | SC |

**Table S2.** The experimental lattice parameters and unit cell volumes of the phases synthesized in the electrical cells.

| *P*6_3_*/mmc* (La, Al) H_10_ | | | | | |  |
| --- | --- | --- | --- | --- | --- | --- |
| Cell | P (GPa) | a (Å) | c (Å) | V (Å^3^) | c/a |  |
| #1 | 146 | 3.7210 | 5.5740 | 33.4 | 1.4980 |  |
| #2 | 156 | 3.7500 | 5.5813 | 34.0 | 1.4883 |  |
| *I*4*/mmm* (La, Al) H_4_ | | | | | |  |
| Cell | P (GPa) | a (Å) | c (Å) | V (Å^3^) | c/a |  |
| #1 | 146 | 2.8304 | 6.0077 | 24.1 | 2.1226 |  |
| #2 | 156 | 2.8301 | 6.0131 | 24.1 | 2.1247 |  |
| #3 | 143 | 2.8911 | 5.9742 | 24.9 | 2.0664 |  |
| #4 | 155 | 2.8450 | 6.0300 | 24.4 | 2.1195 |  |

**Cell #1**

**Figure S1.** SEM image and the EDX analysis results of initial La-Al alloy in cell #1. (a) SEM image and analysis result of the sample ratio. (b) Elemental distribution map of La and Al. (c) EDX analysis spectra.

**Figure S2**. Electrical resistance measurements of La-Al-H sample in cell #1 during compression.


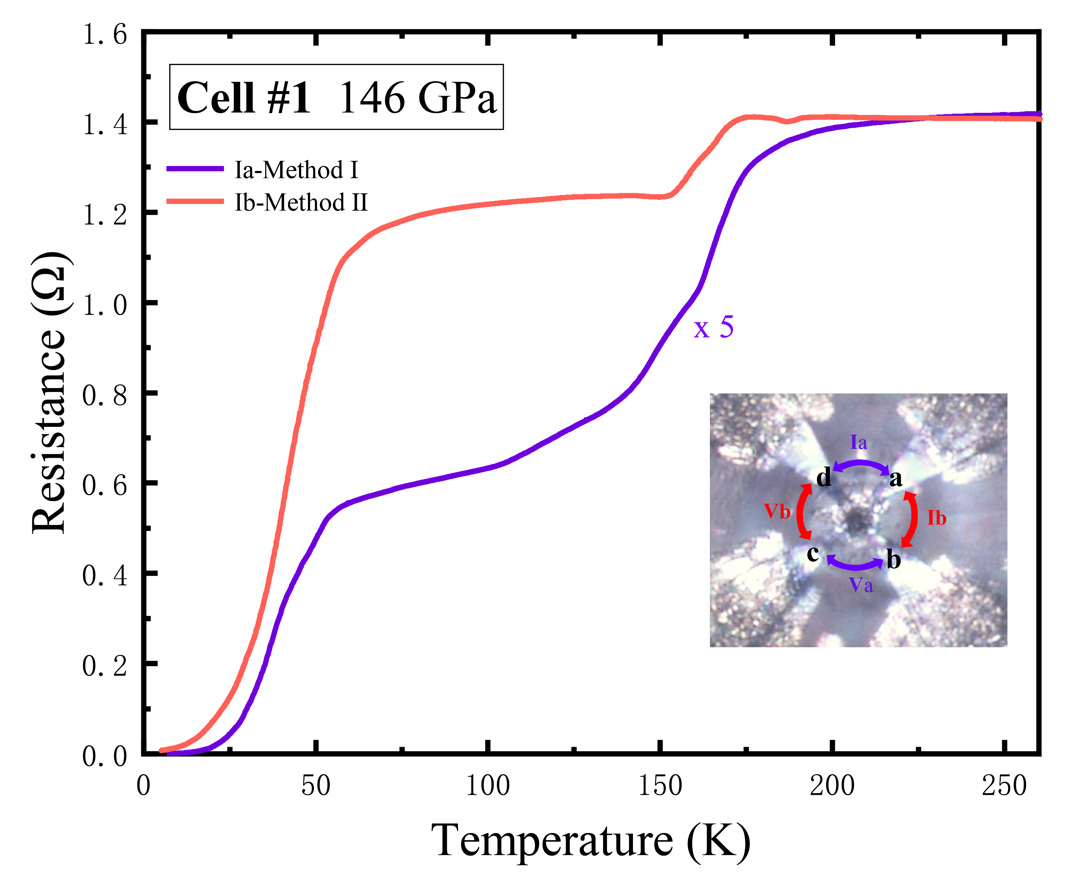


**Figure S3.** Measurement of superconducting transitions of La-Al-H sample in cell #1 at 146 GPa using different electrode combinations. Inset is the four-probe electric measurements photograph of the sample. Method Ia was used for resistance transport measurements and method Ib was used for measuring the isothermal resistance as a function of the field *μ*_0_*H_c_* under the continuous field.


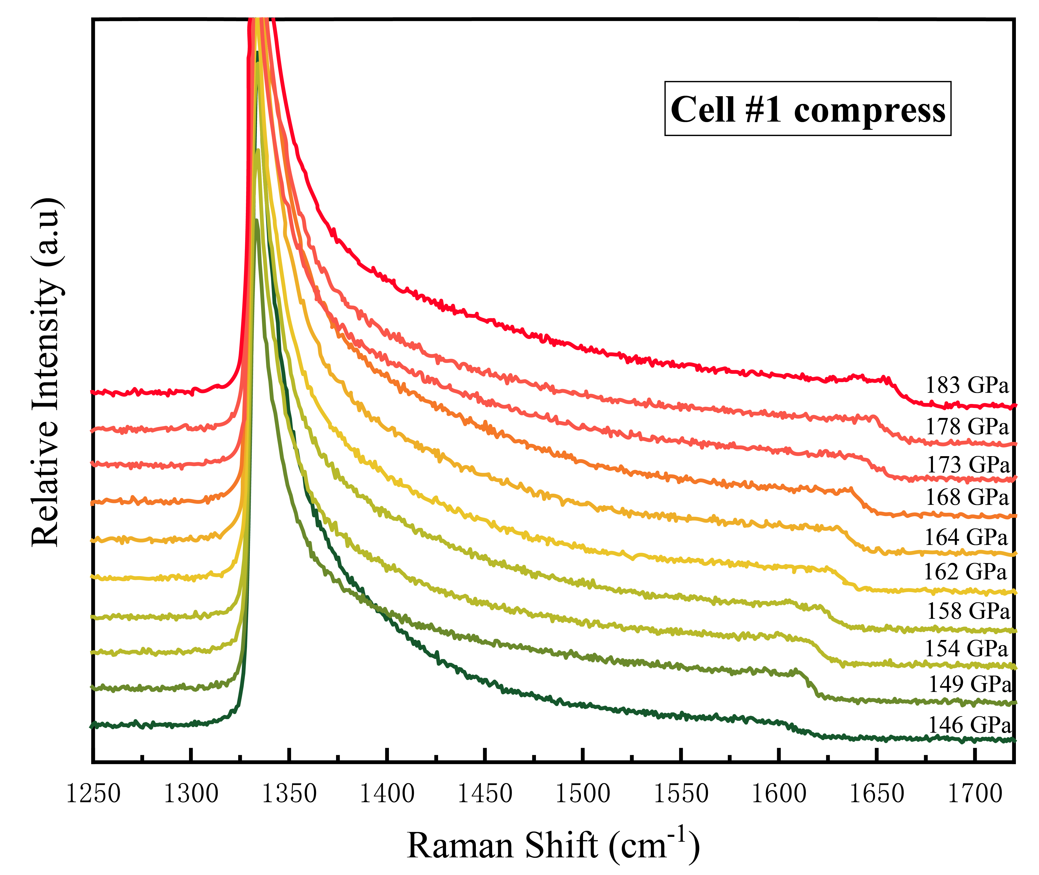


**Figure S4.** The pressure of cell #1 determined by the Raman shift of diamond.

**Cell #2**

**Figure S5.** SEM image and the EDX analysis results of initial La-Al alloy in cell #2. (a) SEM image and analysis result of the sample ratio. (b) Elemental distribution map of La and Al. (c) EDX analysis spectra.


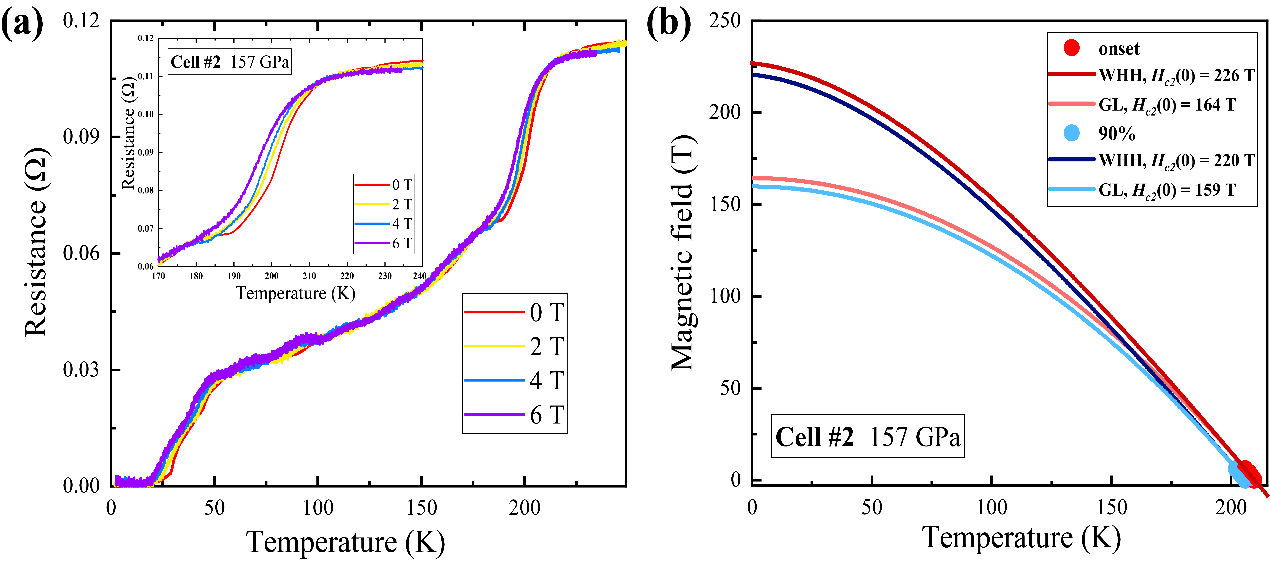


**Figure S6.** Superconducting measurements of La-Al-H sample in cell #2 at 157 GPa in the external magnetic field. (a) In a magnetic field of 0-6 T at 157 GPa. Inset is a partial enlarged view of the data in cell #2. (b) The upper critical magnetic field is estimated by using the WHH [1] and GL [2] models. *T_c_* values are determined by the criteria of the *T_c_*^onset^ and 90% of normal state resistance, respectively.


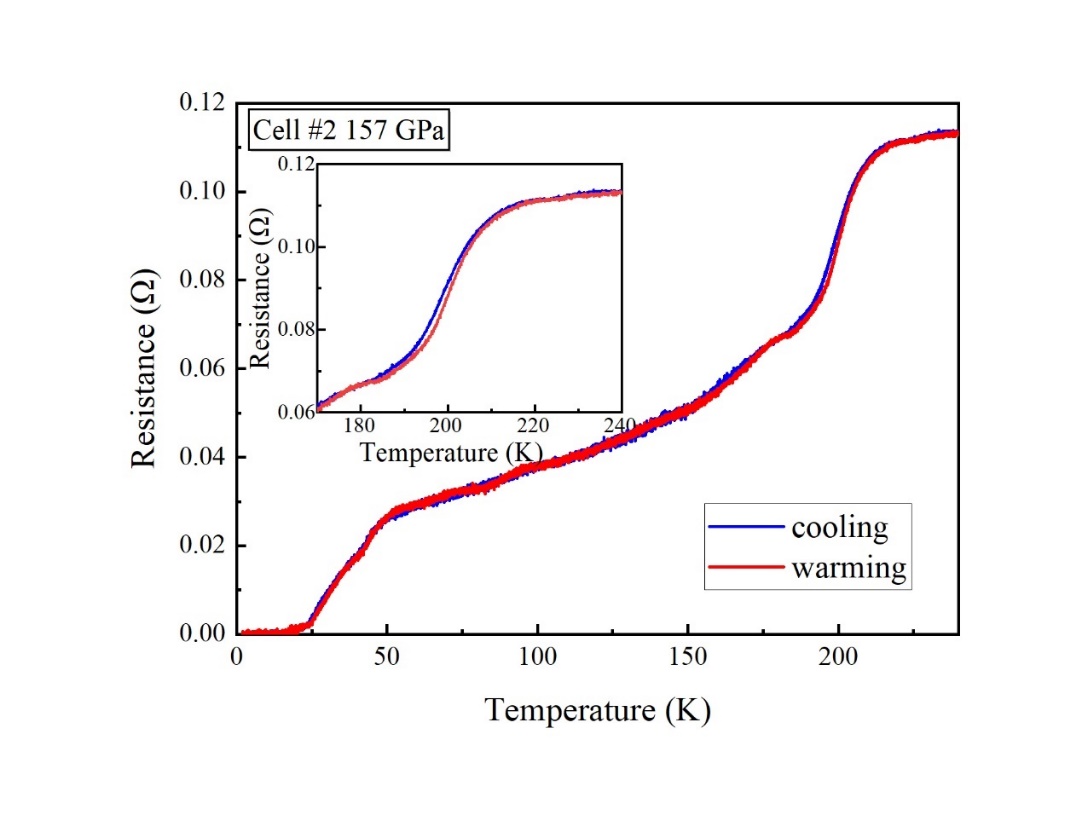


**Figure S7.** Superconducting transitions of cell #2 in the cooling and warming cycle. Inset shows the enlarged superconducting transition of the high-*T_c_* phase. We can see that the *T_c_* of the cooling cycle decreases by about 2 K compared to the warming cycle. The hysteresis in the experiment could be due to a lack of thermal equilibrium.


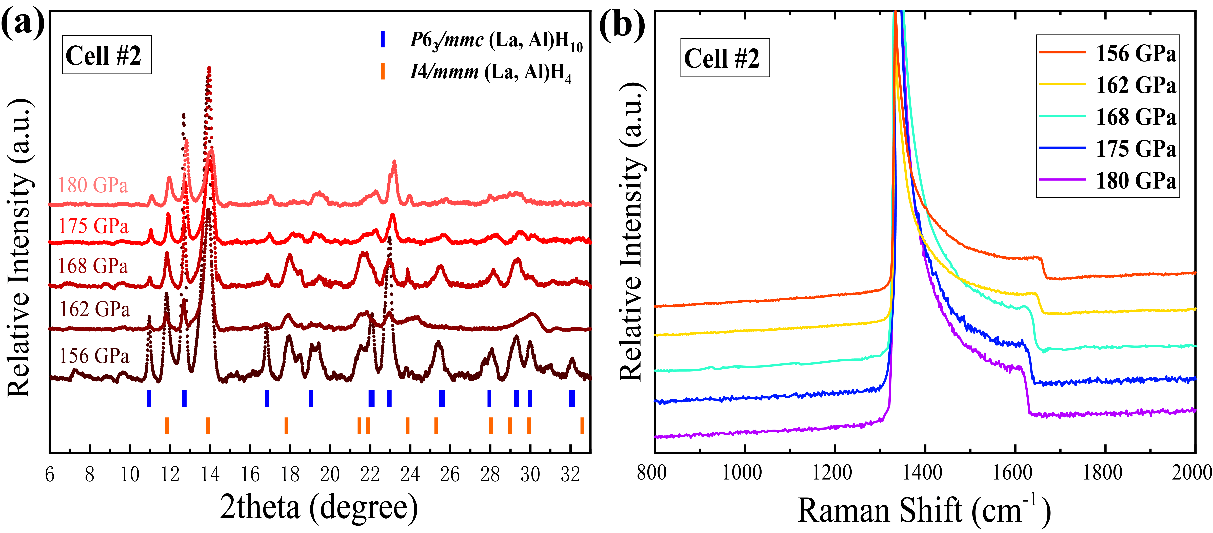


**Figure S8.** Synchrotron XRD patterns of the sample and Raman shift of diamond in cell #2 during compression. (a) XRD patterns (λ = 0.6199 Å) of the sample in cell #2 during compression. The two phases (La, Al) H_10_ and (La, Al) H_4_ are identified. (b) The pressure of cell #2 determined by the Raman shift of diamond.


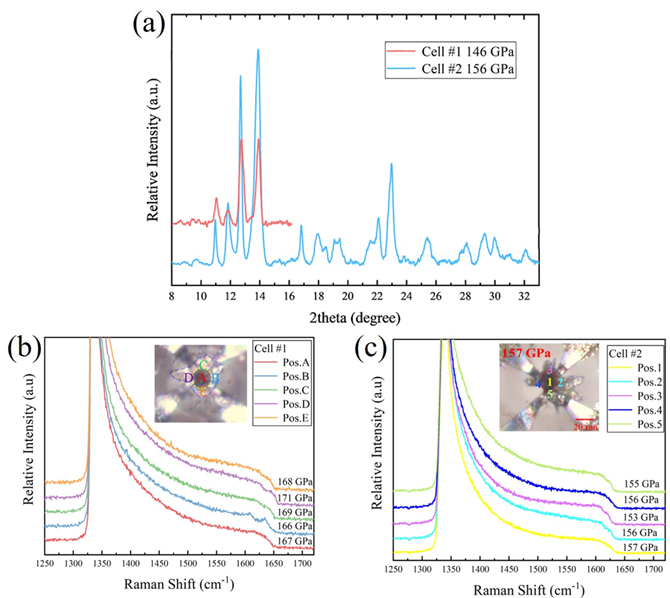


**Figure S9.** (a) The comparison of the XRD patterns for cell #1 and cell #2. The width of the XRD pattern in cell #1 and #2 is nearly same. Raman spectra of diamond for cell #1 (b) and cell #2 (c) at different positions. The pressure gradient is about 5 GPa around the heated samples.

**Cell #3**

**Figure S10.** SEM image and the EDX analysis results of initial La-Al alloy in cell #3. (a) SEM image and analysis result of the sample ratio. (b) Elemental distribution map of La and Al. (c) EDX analysis spectra.


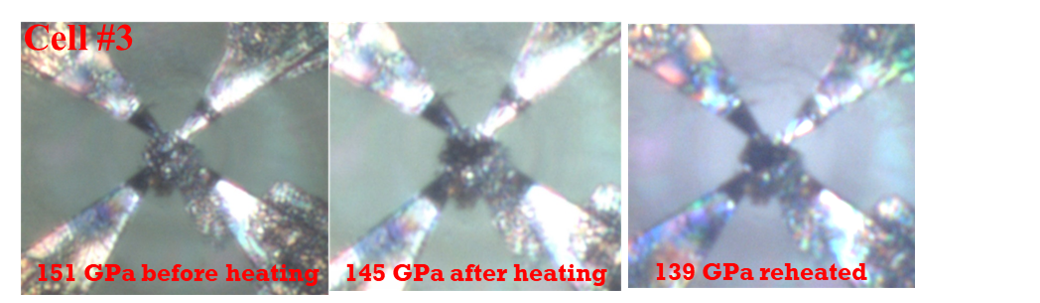


**Figure S11.** The photographs of the sample chamber in cell #3 at selected pressures.


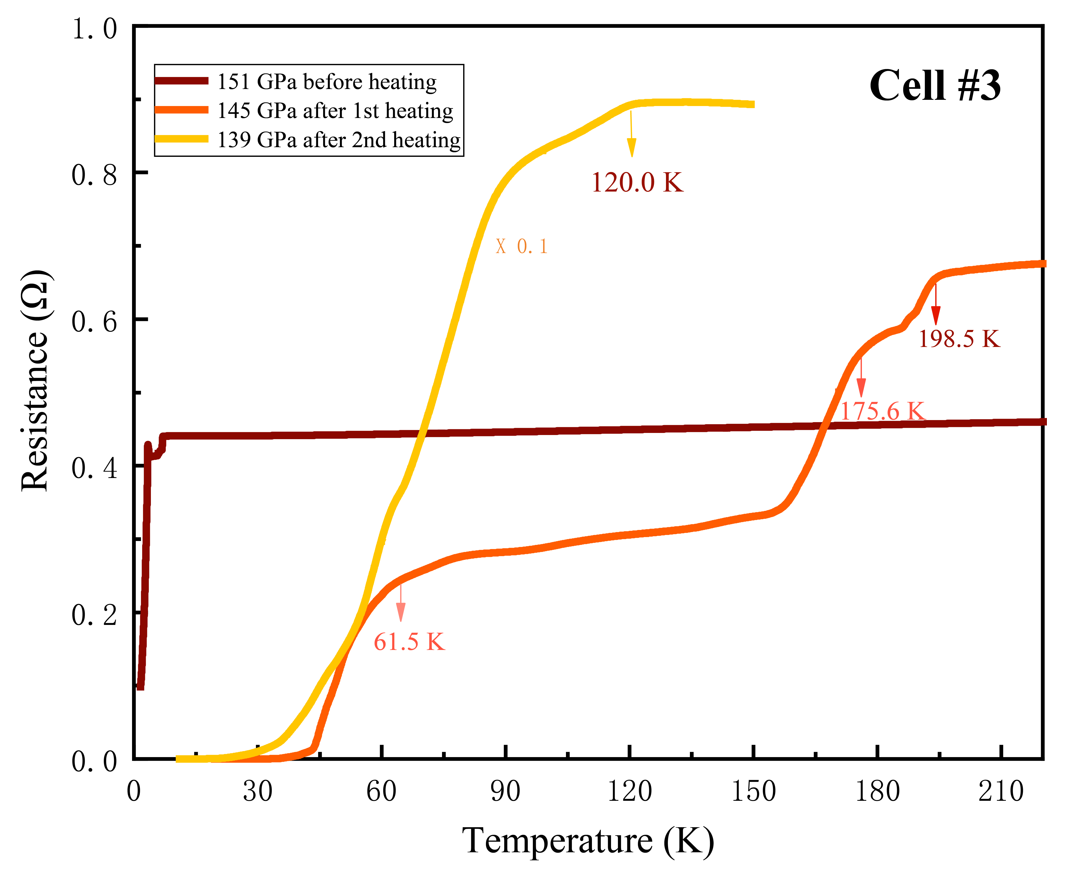


**Figure S12.** Temperature dependence of the electrical resistance in cell #3 before and after the laser heating. The arrows indicated the *T_c_*s. After the first heating, three resistance drops can be clearly seen in cell #3 at 145 GPa with 198.5 K, 175.6 K, and 61.5 K, respectively. However, when the second heating is performed, these two high-*T*_c_ phases cannot be stabilized and decompose into stable hydrides with one superconducting *T*_c_ ~ 120 K.

**Figure S13.** Superconducting transitions of La-Al-H sample in cell #3 in external magnetic fields at different pressures. (a) In the magnetic fields of 0-8 T at 145 GPa after 1st heating. Inset: Upper critical magnetic field estimated using the WHH [1] and GL [2] models. (b) In a magnetic field of 0-8 T at 139 GPa after 2nd heating. Inset: Upper critical magnetic field estimated using the WHH [1] and GL [2] models. *T_c_* is defined at the onset of the resistance drop.


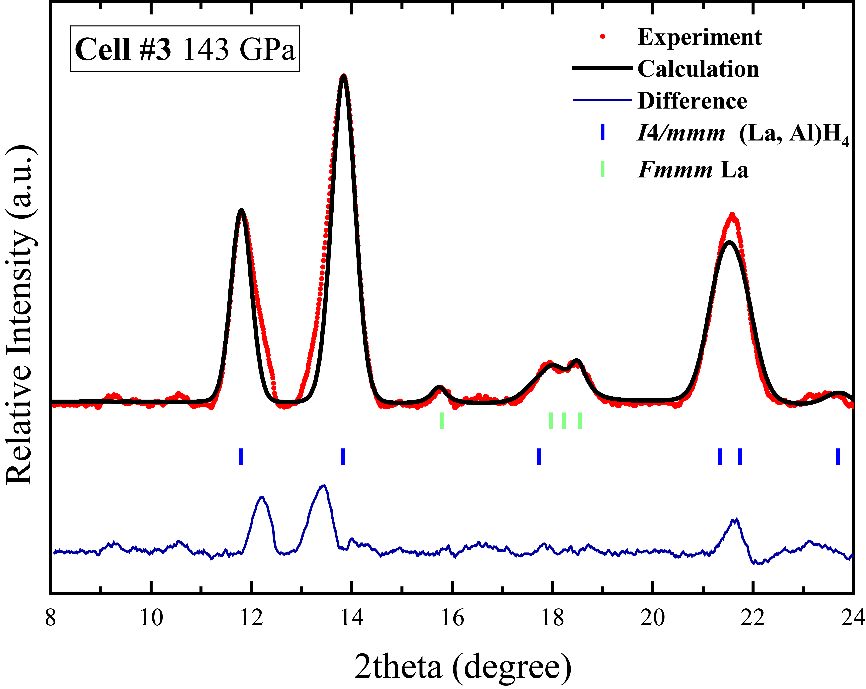


**Figure S14.** Le Bail refinement of the experimental XRD pattern of the sample at 143 GPa in cell #3 after the crack of the diamond. Due to the hydrogen permeability, the diamond begins cracking before the XRD characterization, contributing to the disappearance of superconducting transition with *T*_c_ of 120 K. And only the lower superconducting transition temperature phase (La, Al) H_4_ and element La was left.


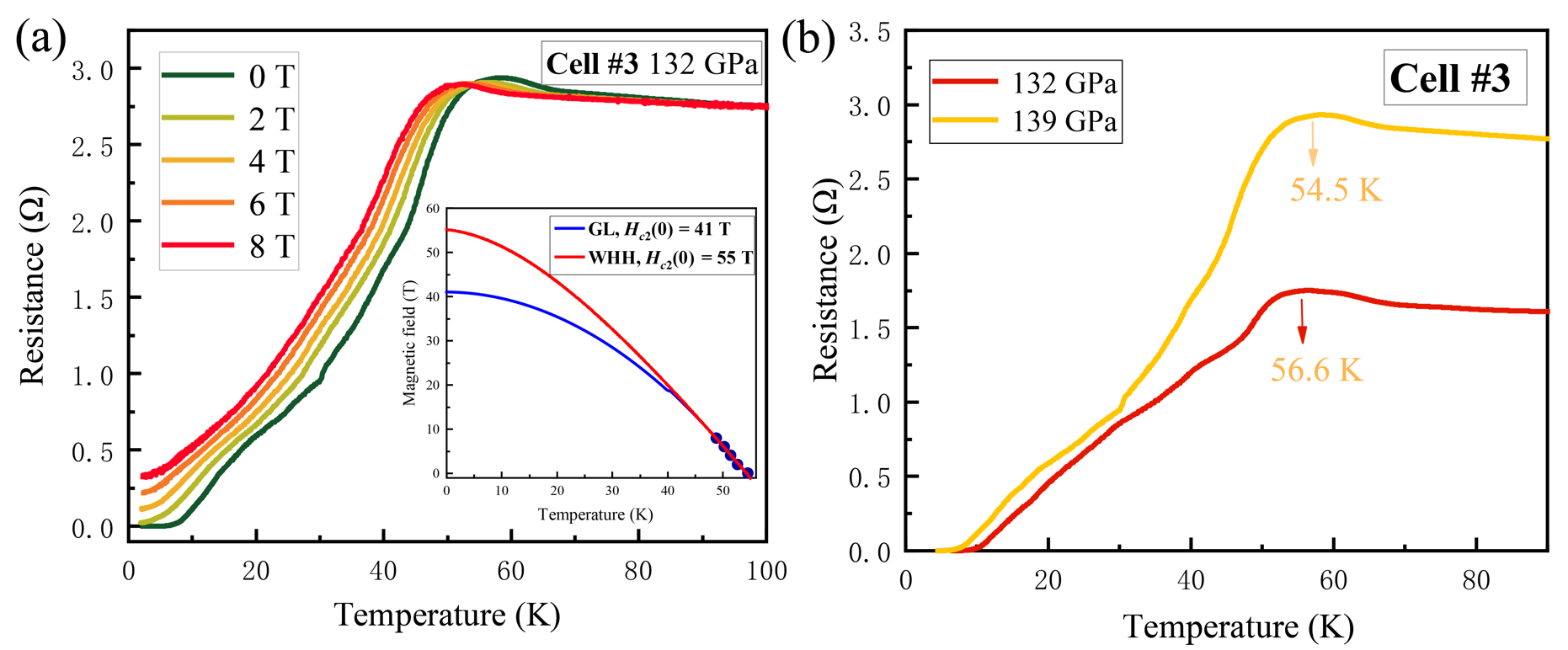


**Figure S15.** Superconducting transitions of sample in the cell #3 after the crack of the diamond. (a) In a magnetic field of 0-8 T at 132 GPa. Inset: Upper critical magnetic field estimated using the WHH [1] and GL [2] models. (b) Superconducting transitions of sample during compression. The arrows indicated the *T_c_*s.


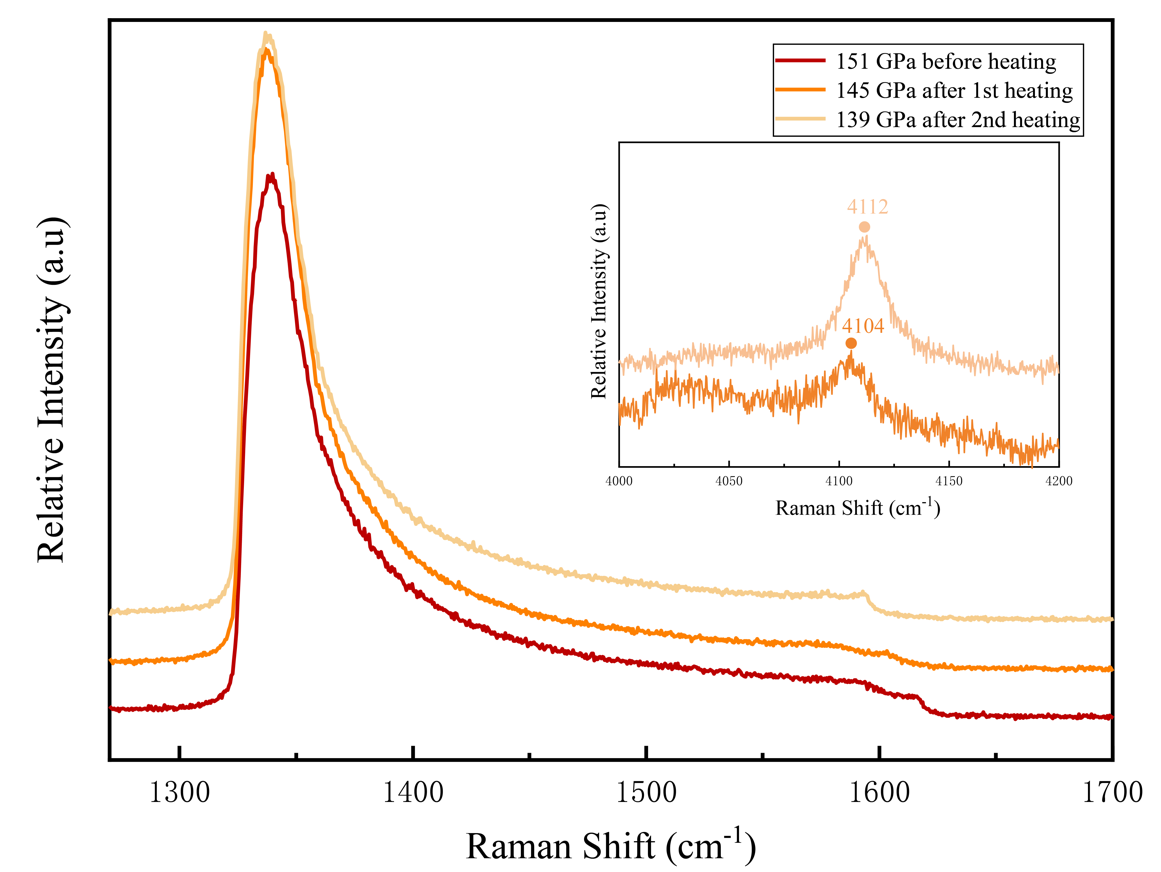


**Figure S16.** The pressure of cell #3 determined by the Raman shift of diamond. The Raman signal of H_2_ is shown in the inset.

**Cell #4**

**Figure S17.** SEM image and the EDX analysis results of initial La-Al alloy in cell #4. (a) SEM image and analysis result of the sample ratio. (b) Elemental distribution map of La and Al. (c) EDX analysis spectra.

**
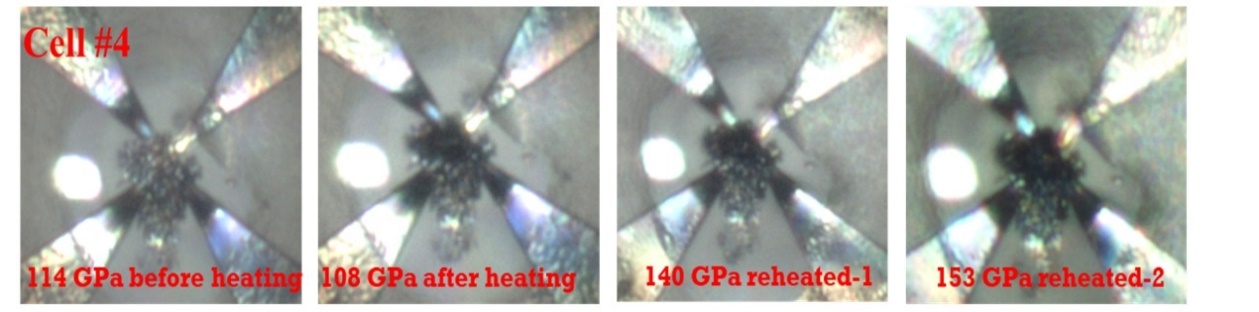
**

**Figure S18.** The sample chamber of cell #4 at selected pressures.


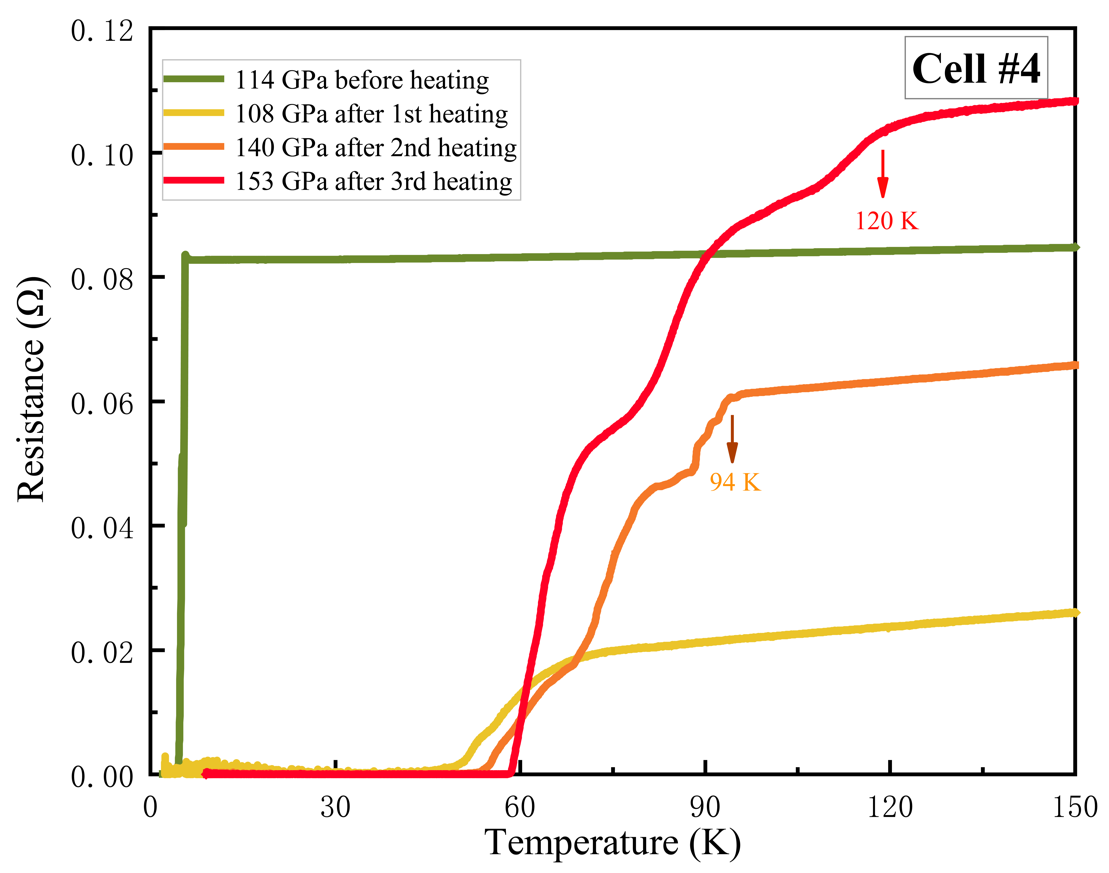


**Figure S19.** Temperature dependence of the electrical resistance in cell #4 before and after the laser heating.

**Figure S20.** Le Bail refinement of the experimental XRD pattern of the sample at 155 GPa in cell #4 after the crack of the diamond.

**Figure S21.** Superconducting transitions of sample in the cell #4 after crack of the diamond in external magnetic field at 165 GPa. (a) In a magnetic field of 0–8 T at 132 GPa. (b) Upper critical magnetic field estimated using the WHH [1] and GL [2] theories.


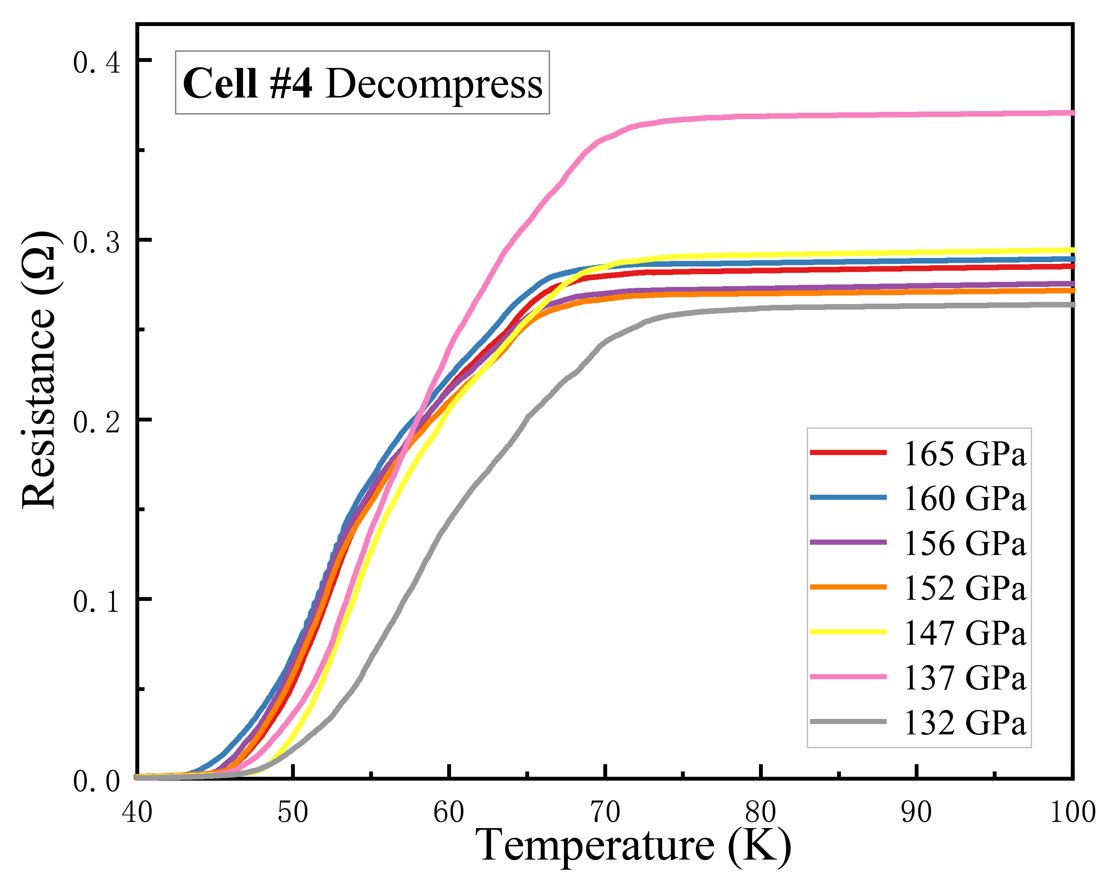


**Figure S22.** Superconducting transitions of sample during decompression in the cell #4.


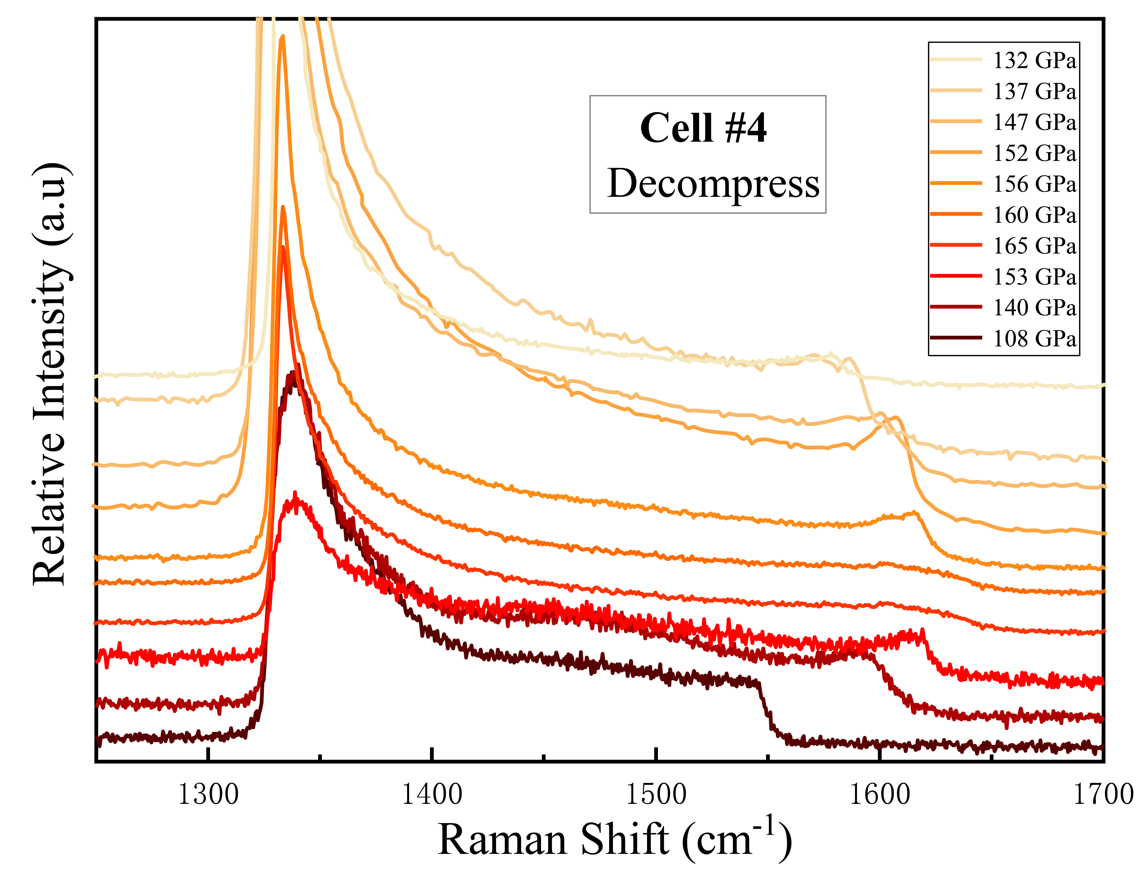


**Figure S23.** The pressure of cell #4 determined by the Raman shift of diamond.

**Cell #5**

**Figure S24.** SEM image and the EDX analysis results of initial La-Al alloy in cell #5. (a) SEM image and analysis result of the sample ratio. (b) Elemental distribution map of La and Al. (c) EDX analysis spectra.

**
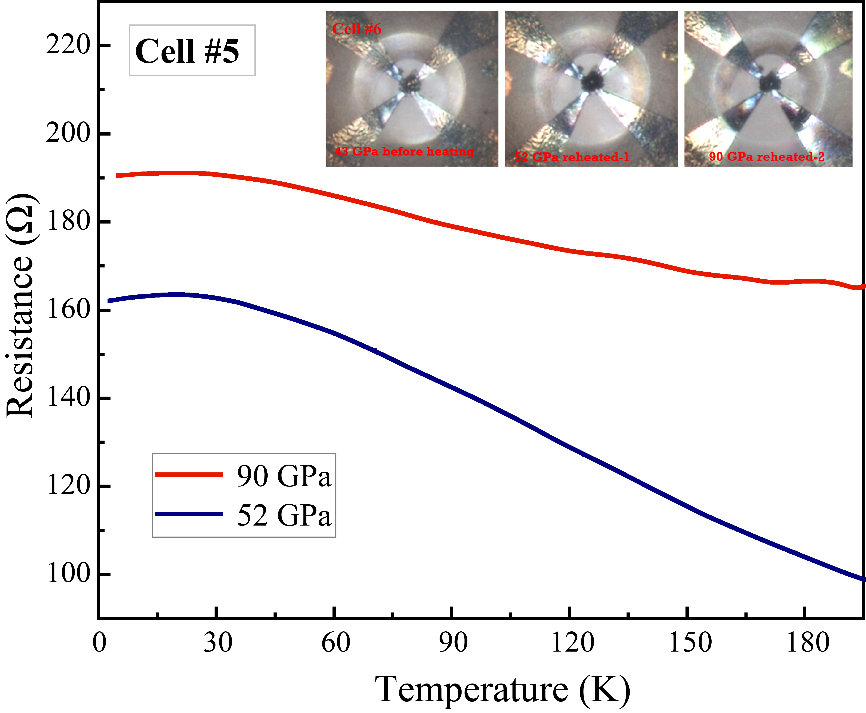
**

**Figure S25.** Electrical resistance of cell #5 at different pressure. Temperature dependence of the resistance in cell #5 after 1st heating at 52 GPa and 2nd heating at 90 GPa during the warming process. Inset: Optical micrographs of the sample chamber in cell #5 before and after laser heating.

**
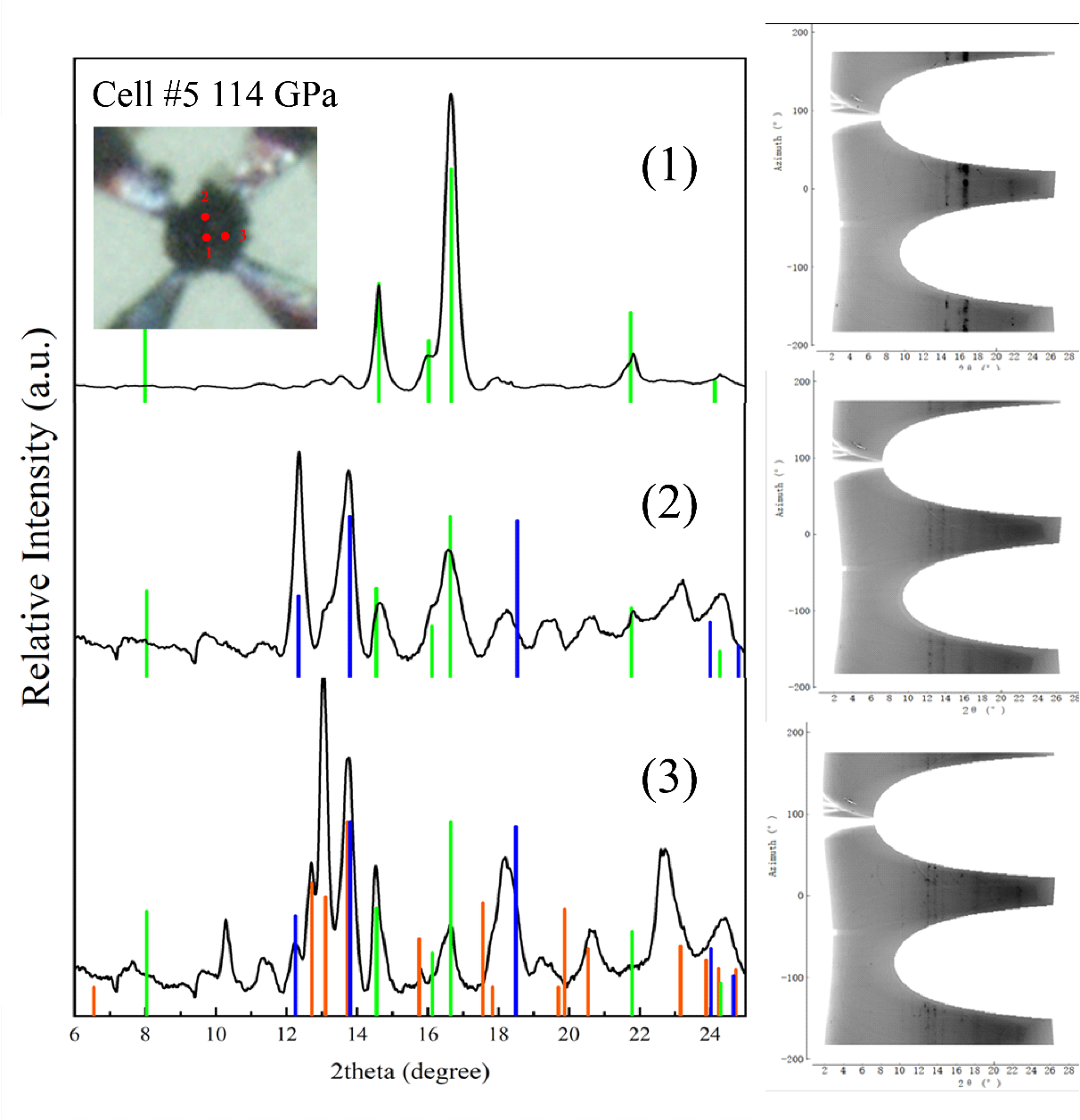
**

**Figure S26**. Diffraction patterns (black curves) at three different locations at 114 GPa pressure, *P*$\bar{6}$*m*2 (La, Al) H_x_ (green marks), *P*6*/mmm* (La, Al) H_2~3_ (blue marks), *Cmcm* (La, Al) H_3_ (orange marks). Inset: Corresponding sample locations for collection patterns.

**Cell #6**

**Figure S27.** SEM image and the EDX analysis results of initial La-Al alloy in cell #6. (a) SEM image and analysis result of the sample ratio. (b) Elemental distribution map of La and Al. (c) EDX analysis spectra.

**
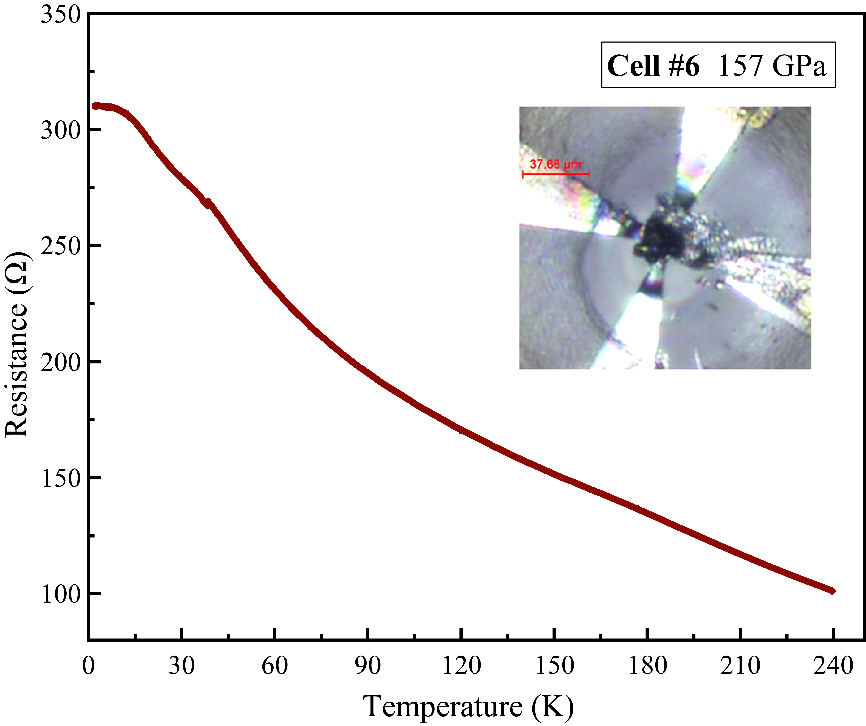
**

**Figure S28.** Electrical resistance of cell #6 at 157 GPa after laser heating during the cooling process. Inset: Optical micrograph of the sample chamber after laser heating in cell #6.

**Cell #7**

**Figure S29.** SEM image and the EDX analysis results of initial La-Al alloy in cell #7. (a) SEM image and analysis result of the sample ratio. (b) Elemental distribution map of La and Al. (c) EDX analysis spectra.


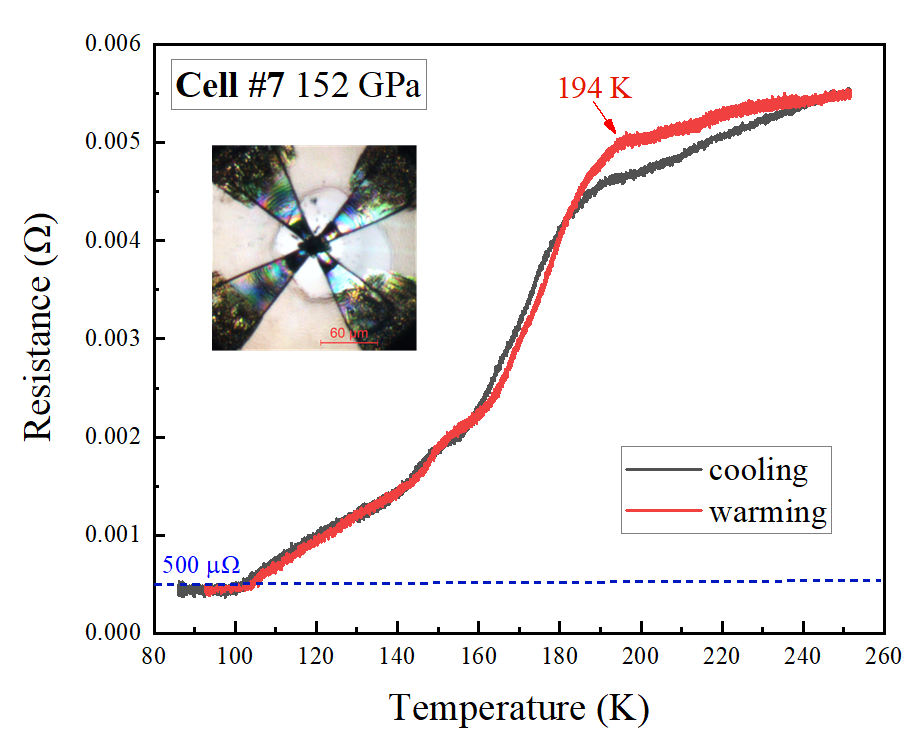


**Figure S30.** Electrical resistance of cell #7 at 152 GPa after laser heating. Inset: Optical micrograph of the sample chamber after laser heating in cell #7.


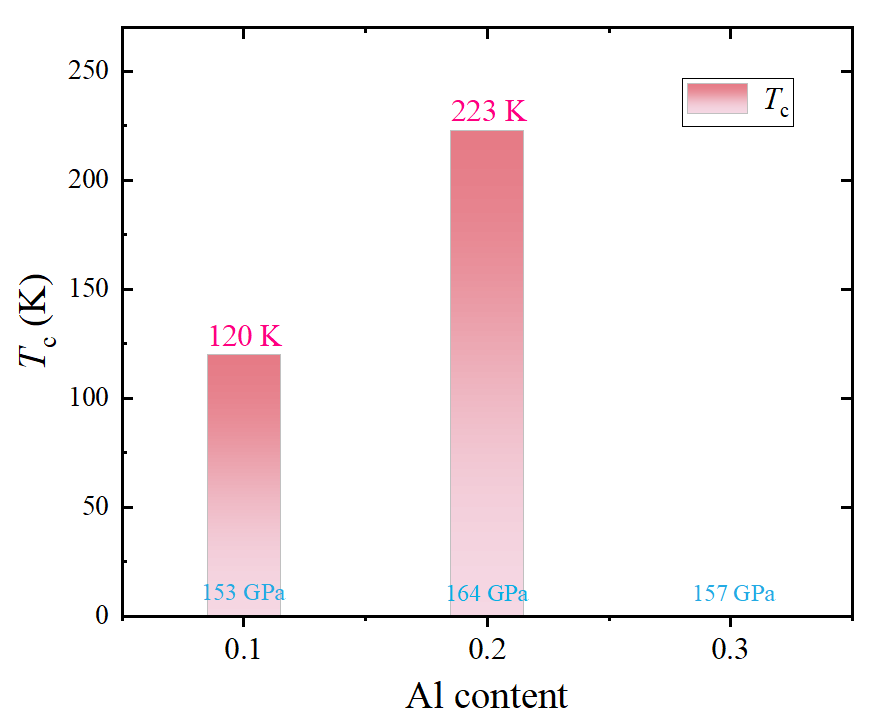


**Figure S31.** The dependence of *T_c_* on the concentration of Al in the La-Al-H system. When the Al content in the initial alloy is high (≥0.3), no *T_c_* is detected in the synthesized samples, and it is more inclined to form a semiconducting phase or some phases with a lower hydrogen content, and when the Al content is appropriate, the optimal superconducting transition temperature of *T_c_* > 200 K will appear.


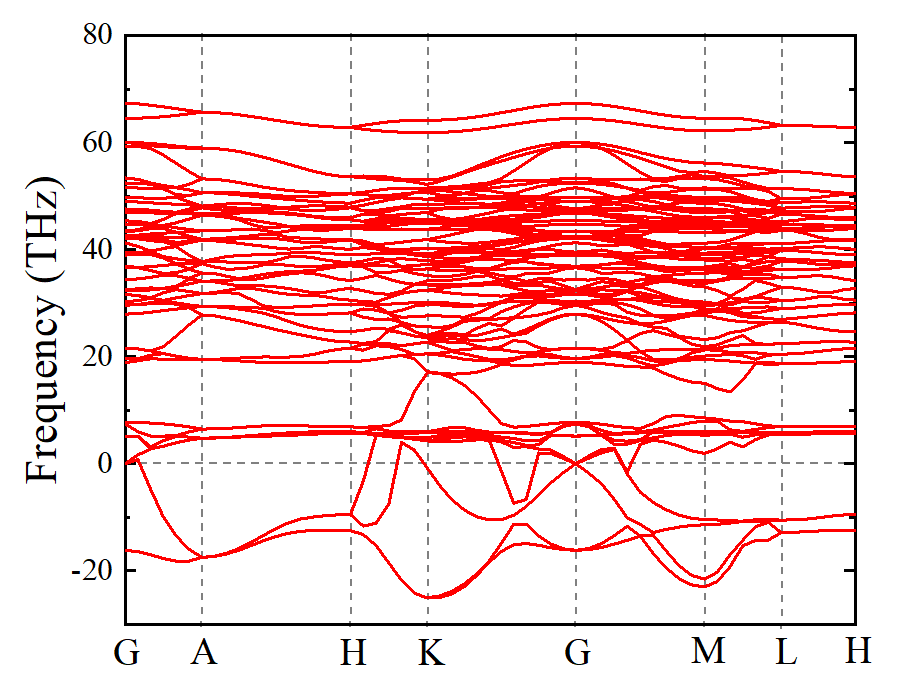


**Figure S32.** Calculated phonon spectra of *P*6_3_*/mmc*-LaH_10_ at 150 GPa. Phonon dispersion calculations for *P*6_3_*/mmc*-LaH_10_ were carried out using the finite displacement method with the PHONONS code [3] in CASTEP code [4]. We used the local density approximation (LDA) local exchange-correlation functional. The kinetic cutoff energy was set to 510 eV, and the Brillouin zone was sampled using the Monkhorst-Pack k-points meshes with a resolution of 2π × 0.07 Å^-1^ to ensure the enthalpy converges to less than 1 meV/atom.

**References**

1. Werthamer NR, Helfand E and Hohenberg PC. Temperature and Purity Dependence of the Superconducting Critical Field, *H_c_*_2_. III. Electron Spin and Spin-Orbit Effects. *Phys Rev* 1966; **147**: 295-302.

2. Ginzburg VL and Landau LD. On the Theory of Superconductivity. In: Ginzburg, VL (ed.) *On Superconductivity and Superfluidity: A Scientific Autobiography*. Berlin: Springer Berlin Heidelberg, 2009, 113-37.

3. Togo A and Tanaka I. First principles phonon calculations in materials science. *Scripta Mater* 2015; **108**: 1-5.

4. Segall MD, Lindan PJD and Probert MJ *et al.* First-principles simulation: ideas, illustrations and the CASTEP code. *J Phys: Condens Matter*. 2002; **14**: 2717-44.
